# Physico-chemical study of complexation of silver ion (Ag^+^) by macrocyclic molecules (hexa-Helicenes) based on statistical physics theory: new description of a cancer drug

**DOI:** 10.1038/s41598-020-67120-4

**Published:** 2020-06-25

**Authors:** Manel Ben Yahia, Mohamed Ben Yahia

**Affiliations:** 1 Physics Department Rabigh College of Science and Arts, King Abdulaziz University, Jeddah, P.O box 344, Rabigh, 21911 Saudi Arabia; 2 Laboratory of Quantum and Statistical Physics, LR18ES18, Faculty of Sciences of Monastir, Monastir, 5000 Tunisia

**Keywords:** Biochemistry, Biophysics, Cancer, Medical research, Chemistry, Engineering, Materials science, Physics

## Abstract

In recent papers, it is found that the silver-[6]Helicene complex can be used as a cancer drug but the interaction silver-hexaHelicene has not yet proven. The idea of this paper is to investigate the complexation process of the [6]Helicene by the silver metal (Ag^+^) using three types of adsorbates. Indeed, the adsorption of silver chloride, silver nitrate and silver sulfide into the sensor films deposited on the QCM electrode are measured at three temperatures (293–333 *K*). Films of the [6]Helicene were deposited on the QCM resonators using spin coating method in order to obtain uniform and homogenous sensor surface. Experimental results indicated that the [6]Helicene can form a stable complex with the silver ion and that the AgCl is the appropriate adsorbate for the complexation achievement. Actually, an advanced modeling analysis by means of statistical physics adsorption models is applied to explore the new vision of the complextion system. The values of the models parameters are deduced from fitting the experimental data with the developed models. They result in confirming the experimental findings by comparing the complexation energies of the three examined systems. In particular, for the silver nitrate, the Van-der-Waals parameters explained the isotherms drop at high concentration through the lateral interactions between the adsorbates. The adsorption energies analysis showed the highest interaction AgCl-[6]Helicene. Density functional theory (DFT) simulations showed that chemical bonds take place during the adsorption of silver chloride on hexaHelicene which confirms that the [6]Helicene can function as a chiral molecular tweezer of the univalent cationic silver.

## Introduction

In recent years, several experimental and theoretical examinations have been carried out on Helicenes and related molecules including the determination of their racemization barriers^1^, aromatic character and non-linear optical properties^2,3^. In addition, recent works emerged the transition metal-based helicenes as novel attractive chiral molecules for various reasons. Firstly, metals are powerful templates for assembling π-conjugated ligands into well defined molecular structures; simply by using the fundamental ideas of coordination and organometallic chemistry^4^. Secondly, novel characteristic can be shown when metallic particles are grafted onto π-helical structures^5–7^. In particular, these chiral molecules are involved in several research fields such as molecular recognition^5,8,9^, supra-moelcular chemistry^1^ and catalysis of transition metal^2^ because of their extraordinary geometry and their electronic properties^3^. Finally, the complexation of Helicenes by metals suggests the development of future devices in optoelectronics as chiral waveguides and we can think of planning new materials for the modulation of information in telecommunications^5^.

Moreover, It has been shown that thin layers of adsorbed ions (Li^+^, Mg^2+^, Fe^2+^, K^+^, Na^+^…) based functionalized heptaHelicene or macrocycle molecules (e.g porphyrins) can be obtained on solid support of quartz crystal^10–12^. The QCM strategy includes the preparation of thin-film of composite doped on quartz crystal which is demonstrated to be highly reproducible and permits outstanding control over the adsorbed film thickness. A wide range of strategies have been adopted to cover electrode surface with similar composites: direct adsorption, incorporation inside PVC or other inert polymer matrices, polymerisation–deposition of the species of interest on the electrode surface^13^. Recently, the quartz crystal microbalance has been demonstrated more powerful than the other methodologies^14^. It involves the spin coating of Helicene volume on the quartz electrode to cover the overlapping electrodes portion. The objective is to acquire appropriate films which should be homogeneous over the whole surface of the quartz crystal^15,16^. Films formed in this way have several virtues: first, they are mechanically stable and tolerant of different solvents and reaction conditions used for common organic transformations^15^; second, they are adequately well ordered that globally most of the complexing Helicenes are attractive candidates for this application because they have been demonstrated to be dynamic adsorbent in the complexation processes^16^. Interestingly, it should be noted that the QCM technique was devoted for the achievement of experimental adsorption isotherms by identifying the complexed masses of metallic ions for a wide range of concentration under various temperatures^10–12^. The initial success of this experimental approach has encouraged us to study the complexation of silver ions on hexaHelicene and to investigate the microscopic properties of the formed complex. Thus, it appears that the complex Ag^+^-Helicene can serve as a drug in the fight against cancer^17^ but the adsorption of silver ion on Helicenes does not seem to be significantly studied despite the importance of this complex in the medical fields. This motivated us to study the experimental (also theoretical) possibility of hexaHelicene acting as a complexing sensor of the silver ion (Ag^+^). Hence, a study of adsorption at the solid-liquid interface is required^18^.

**Figure 1:**
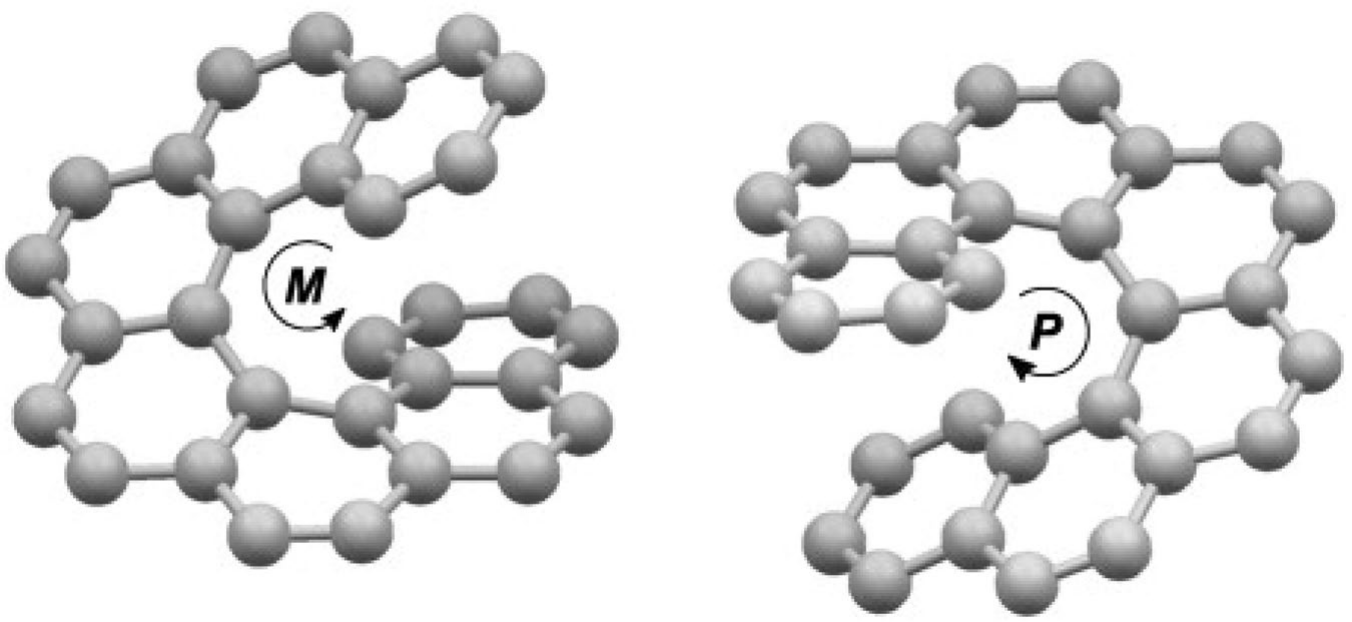
Representation of the two spatial conformations (enantiomers) ***M*** and ***P*** of hexa-Helicene molecule.

**Figure 2:**
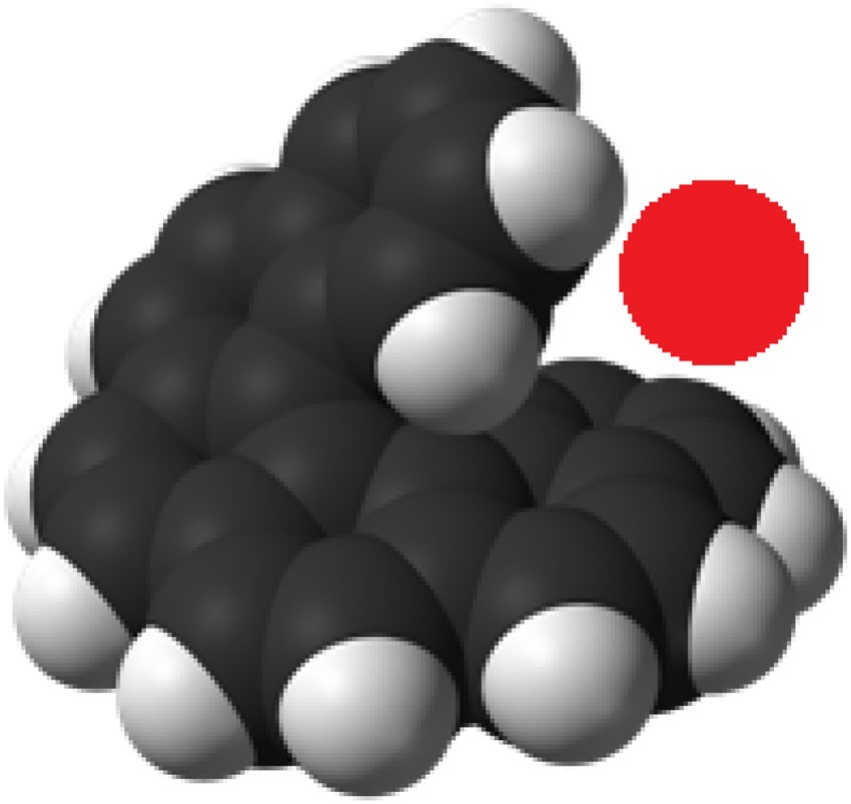
Profile of hexaHelicene molecule trying to ‘devour’ a silver metal.

In reality, hexaHelicene is an interesting member of the polycyclic aromatic hydrocarbon family called carbo-Helicenes^1,19^. It is characterized by its helical geometry offering rise to two enantiomers (Fig. 1). Six benzenoid rings offer ascent to scaffolding that finishes a total turn of the helix. In addition, the terminal rings of these Helicenes appear to be cofacials. The profile of these molecules shows the two rings of benzene overlapping like jaws of a crocodile (Fig. 2). So, it is intriguing to check if this small molecule has the adequate flexibility to complex the cationic metals^5,20^.

Actually, several examinations indicated that the heptaHelicene can complex the alkali metals (K^+^, Na^+^, Cs^+^, Li^+^…)^10,21,22^. However, it is exhibited that the binding of the silver (I) particle in the focal cavity of [7]Helicene shows that the counter ion is feebly coordinated^23^. In such coupling mode, the [7]Helicene is working as a chiral molecular tweezer but the bond is not strong and the complex formed is not stable. For this reason, in this paper, we choose to use the hexaHelicene as adsorbent material seeking for formation of more stable complex. At this point, it should be noted that up to now, a cation-π complex with [6]Helicene has not been proven^24^.

The study of the insertion mode of alkali silver into [6]Helicene cavity is the subject of this work. Indeed, the initial segment aims to achieve the adsorption isotherms of silver chloride, silver nitrate and silver sulfide on [6]Helicene layers using the quartz crystal microbalance technique^16,25^. The second part is committed to the modeling of these isotherms using the adsorption models developed through statistical physics treatment^11,12,21,22^. This modeling permits the determination of the best adsorbate regarding its stability and reactivity in a practical industrial application through the comparison of the adsorption capacities. The choice of the adsorbate-adsorbent couple is also checked by applying the density functional theory for interaction energy calculation^26^. The application of the DFT method permits foreseeing the most plausible structure of the resulting complex^26^.

## Determination of Experimental Isotherms

**Figure 3:**
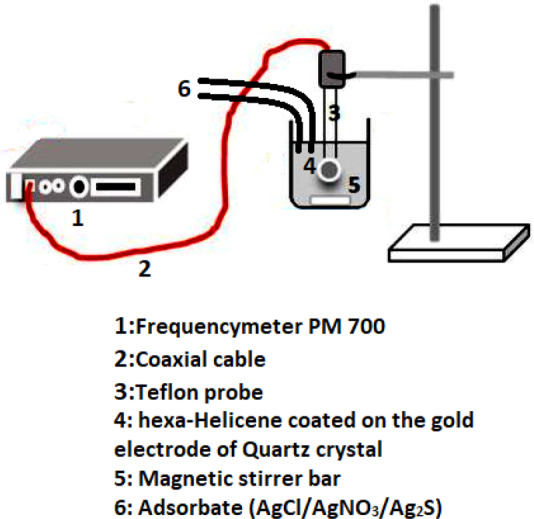
Experimental setup of Quartz Crystal Microbalance strategy devoted for the achievement of experimental adsorption isotherms of silver chloride, silver nitrate and silver sulphide on hexa-Helicene molecules.

In this experimental section, the quartz crystal microbalance (QCM) strategy (Fig. 3) is devoted to the detection of silver (I) on modified electrodes containing [6]Helicene^15,17^. As a methodology, the QCM developed a solution measurement capability in largely analytical chemistry and electrochemistry applications due to its sensitive solution-surface interface measurement capability. The past decade has witnessed an explosive growth in the use of the QCM technique to the investigation of a wide scope of molecular systems at the solution-surface interface since the experimental setup directed for the achievement of experimental isotherms is a simple and effective mass sensing method based on the piezoelectric characteristic of the quartz crystal. Any change of mass at the surface of the quartz crystal will be preciously identified by the microbalance apparatus^27^. In fact, this technique requires cleaning of the quartz crystal and then covering the gold electrode with thin layer of Helicene.

### Preparation of adsorption cell

The commercial quartz crystal was protected by resin layer which should be removed using a well-known cleaning protocol^28^: the material was rinsed with acetone and deionizer water followed by drying. Then, it was cleaned with a Piranha solution at room temperature for 1 to 5 minutes. After this cleaning treatment, the crystals were completely rinsed with deionizer water and ethanol, and then dried by applying high purity nitrogen to remove any remaining water. Note that the crystals used in the experiments are polished with a fundamental resonance frequency of 5 *MHz* and have a diameter of 2.54 *cm*.

Then, a modification of the active surface of the crystal was carried out by coating the hexaHelicene adsorbent: 30 *µl* of adsorbent was deposited at 3000 *rpm* for 1 *min* onto the quartz crystal surface. The covered crystals were then dried at 100 *°C* for 2 hours. After drying, the resonance frequency of the crystal coated with the adsorbent was measured. The difference between the frequencies of the virgin and the coated crystal was calculated. By the intermediate of this frequency difference, we calculated the mass of the deposited adsorbate. The design of such chemically modified electrodes is to control if the Helicene molecules were capable of forming stable complexes with silver ions in the close proximity of the electrode surface. Once the adsorption cell is prepared, we proceed with the isotherms measurement.

### Measurement of experimental adsorption isotherms

In the reactor filled with the buffer solution (*V*_*s*_ = 130 *ml*), the Adsorption cell ([6]Helicene coated on quartz crystal) was immerged until the adjustment of the frequency. Once the QCM probe was immersed in water, the hydrostatic pressure caused a change in the resonant frequency. This frequency (*F*_0_) was measured by a Maxtek frequency-meter PM700 and was taken as reference for our measurement.

Then, stock solutions of adsorbates AgCl, AgNO_3_ and (Ag)_2_S were prepared. Adding a volume *V*_*ad*_ to the initial volume *V*_*s*_ permitted to obtain concentration of solutions ranging from 10^−7^*M* to 1.6.10^−2^*M*. If we note by *c*_0_ = 10^−4^*mol/l* the concentration of the stock solution of the adsorbate already prepared and we need to obtain a final concentration in the reactor *c*_*f*_ = 10^−7^*M*, the additional volume was determined as follows:

1 $${V}_{ad}=\frac{{c}_{f}.{V}_{s}}{{c}_{0}}$$

The added volumes for each concentration were calculated according to the last equation. So, volumes of adsorbate solution were injected using a micropipette in order to increase the adsorbate concentation in the reactor. For example, to obtain the concentration 10^−7^*M*, 100 *µl* of adsorbate solution (10^−4^ *M*) was injected into the reactor followed by magnetic stirring for 5 *min* to homogenize the solution. After each addition, the microbalance apparatus displayed the resonance frequency associated with the new concentration. This step was repeated according to the number of performed injections.

Furthermore, it is necessary to quantify the quartz resonant frequency by assigning the different parameters to the corresponding frequency in order to correctly exploit the experimental results. In fact, once the sensor is immersed in a liquid, the model which describes the response of a quartz is called the Kanazawa-Gordon model whose change of oscillation frequency is affected by the properties of the liquid which are the density and the viscosity of the solution, by the temperature, by the hydrostatic pressure of the solution and by the roughness of the adsorbent surface^29–32^. Then, the total frequency is written as follows:

2 $$\Delta f={F}_{i}-{F}_{0}=\Delta {f}_{m}+\Delta {f}_{T}+\Delta {f}_{p}+\Delta {f}_{r}+\Delta {f}_{\eta ,\rho }$$

where, ∆*f*_*m*_ describes the influence of the mass change, ∆*f*_*T*_ is the temperature effect, ∆*f*_*p*_ is the pressure effect, ∆*f*_*r*_ is the roughness effect and ∆*f*_*η,ρ*_ represents the liquid properties effects described by Kanazawa which are due mainly to the variation of the viscosity and the density of the solution.

In our examination, the temperature, the pressure and the roughness were irrelevant because the experimental measurements were achieved in a thermostatic cell at fixed temperature using polished quartz. Moreover, low impacts of solution properties were noted so they were not taken into account^31^. It can be concluded that the frequency change was mainly due to the deposited mass of adsorbate (∆*f* = ∆*f*_*m*_). In this case, the adsorbed mass was determined from the Sauerbrey’s relation which assumed that for small masses changes, the added mass can be treated as an additional mass of the quartz^33^. Thus, the frequency-mass relation is described as follows^33^:

3 $$\Delta f=-\,{C}_{f}.\Delta m$$

where, *C*_*f*_ is the Sauerbrey’s constant *(Hz*.cm^2^/µg*)*, *∆f* is the frequency shift *(Hz)* and *∆m* is the additional mass deposited on the quartz (µg/cm^2^).

**Figure 4:**
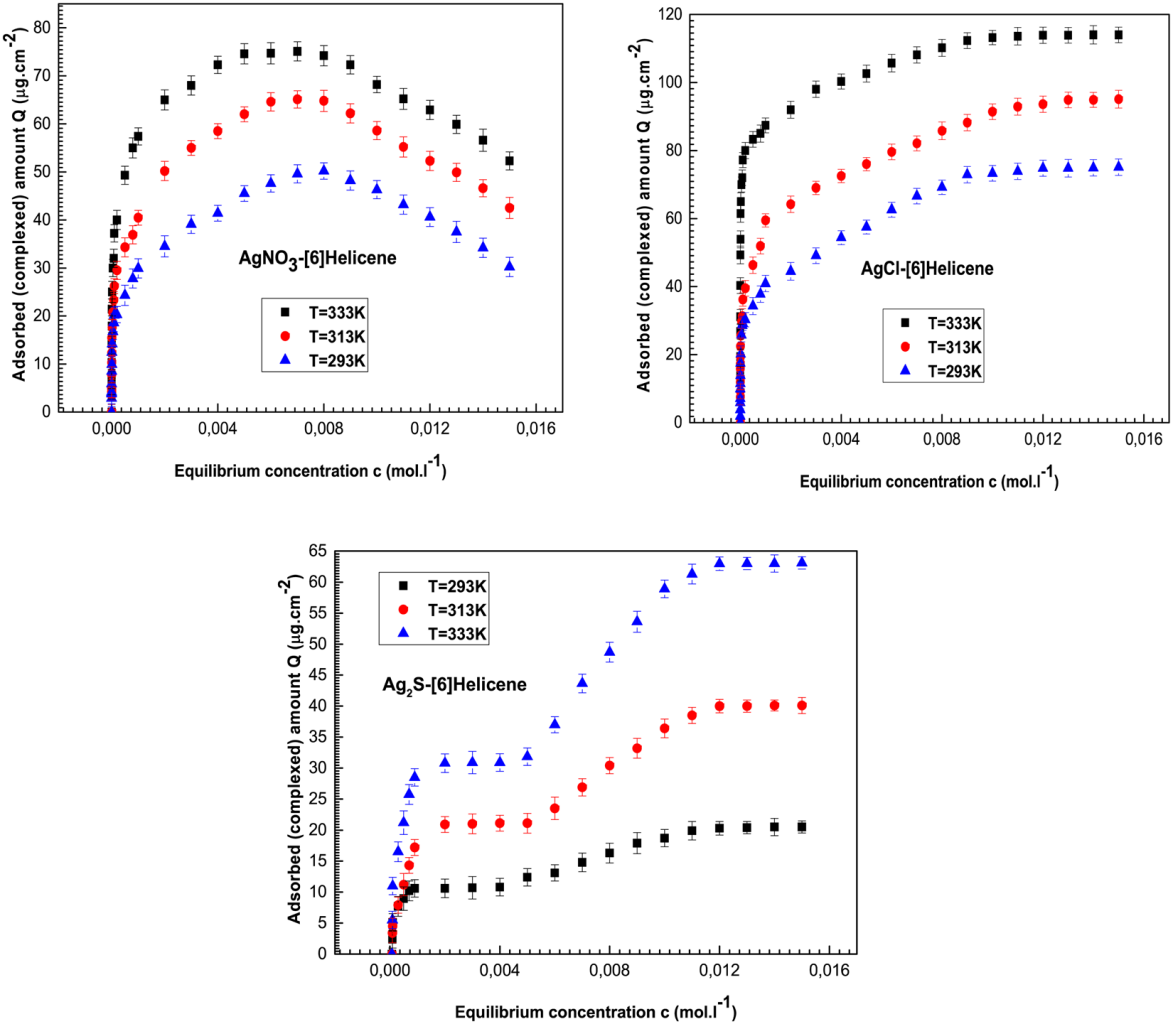
Complexed quantity per unit of surface area ***Q*** **(*µg.cm***^***−2***^**)** describing the adsorption of silver chloride, silver nitrate and silver sulfide on hexa-Helicene molecules given at 293, 313 and 333 *K*.

We can finally deduce the complexed quantity per unit of surface for each concentration and afterward, we obtain the equilibrium adsorption isotherms of silver chloride, silver nitrate and silver sulfide onto hexaHelicene which are illustrated in Fig. 4 at three different temperatures.

### Results of QCM measurements

In view of the experimental isotherms profiles, we note the following results:

Firstly, the experimental adsorption isotherms indicated that the silver particles are captured by the [6]Helicene molecules at various temperatures. Therefore, it is demonstrated that the [6]Helicene can function as a sensor of silver particles.

Secondly, one can see that the behaviors of isotherms curves are totally different for the three adsorbates, despite all experimental data were accomplished under the same conditions and using the same adsorbent (hexaHelicene). One can conclude that the isotherms profiles depend on the adsorbate type which constitutes the dominant factor of the complexation phenomenon. Moreover, by looking at the adsorbed amounts of the three adsorbates, we hold that silver chloride presents the highest adsorption capacities and in this way it was the best adsorbate for Helicenes complexation.

Thirdly, we note that an expansion in the temperature causes an increase in the adsorbed amount for all adsorption systems. So, the temperature effect indicates that the Helicene complexation is an endothermic process whatever the adsorbate is.

Finally, it is noticed that the silver chloride isotherms present one saturation level indicating that only one layer of cationic ions Ag^+^ is adsorbed onto the solid support of [6]Helicenes while the chlorine ions (Cl^−^) stay as spectator ions in solution and don’t impose any impact on the complexation mechanism. At high adsorbate concentration, we can see a stable saturation state for the AgCl which means that all hexaHelicene sites are occupied and that the AgCl-[6]Helicene interaction is strong so cutting this link is almost impossible. For silver nitrate adsorption, one saturation level followed with a drop is observed at the three temperatures which suggests the presence of an irreversible phenomenon after the saturation state. This can be explained by the fact that the AgNO_3_- [6]Helicene bond is weak so the silver ions can escape from the crocodile jaws ([6]Helicene cavity) at any time which leads to the decrease of the adsorbed quantity at high concentration. The isotherms profile of silver sulfide is totally different from the others. Two saturation states are shown for the adsorption isotherms of Ag_2_S on hexaHelicene which leads to estimate the formation of two adsorbed layers. So, the use of silver sulfide leads to conclude that layer by layer adsorption^13,34^ takes place and that the anionic sulfur ions have an impact on the complexation mechanism: the [6]Helicene acts as a complexing adsorbent of the silver Ag^+^ and after the formation of the first layer, the adsorption of the anionic ion S^2−^ takes place until the total saturation at high concentration.

According to these explanations, one can conclude that silver chloride is the suitable material for the fabrication of a stable complex silver-hexahelicene.

In the next section, statistical physics treatment is proposed for modeling analysis of adsorption isotherms. The goal is to elaborate a theoretical description of these experimental results at the ionic scale.

## Statistical Physics Analysis

In this section, the microscopic investigation of the three complexation systems is carried out by adopting advanced statistical physics models. This approach has been recently used with great success^10,21,22,26^ in adsorption isotherms modeling.

### Development of advanced models

The experimental isotherms indicated that the adsorption of silver chloride and silver nitrate take place with single-layer formation whereas, L.B.L double-layers formation is concluded for the silver sulfide adsorption. Moreover, the silver chloride and the silver sulfide isotherms show stable saturation levels with no disturbances. These isotherms can be interpreted by the classical single-layer and double-layers models which are established on the basis of the ideal gas approach. However, one saturation level followed with a drop is observed for the silver nitrate isotherms. According to previous studies, this decrease in the adsorbed quantity after saturation is mainly due to the lateral interactions between the adsorbate particles at free state^10,21,35^. This description requires the use of the chemical potential of real gas in which the lateral interactions are introduced.

Actually, the development of adsorption models using the statistical physics treatment requires the following assumptions^22,35,36^:

1. The use of the grand canonical ensemble is necessary to assess the variation of adsorbed ions number in relation with the concentration.
2. Only the translation degrees of freedom are taken into account. All the other internal degrees of the particle (rotational, vibrational, electronic and nuclear) are neglected because the investigated metallic ion in this paper is monoatomic (Ag^+^).
3. It is known that the [6]Helicene has two spatial conformations ***M*** and ***P*** **(**Fig. 1**)**^13^. So, it is assumed that the receptor sites of the adsorbent are divided into two types and that a self-adsorption competition of the silver metal takes place between the two kinds of receptor sites, each with its relating energy level. Therefore, the statistical physics models developed in this paper are the single-layer model with two energy levels and the double-layers model on two types of sites.

#### Single-layer adsorption

For the single-layer adsorption model, it is assumed that the silver particles followed a monolayer adsorption process. They are adsorbed on the first adsorbent type *H*_*M*1_ with the first energy (-*ε*_*1*_) while the adsorption on the density *H*_*M*2_ of the second type of sites is carried out with the second energy level (-*ε*_*2*_). Then, the partition-function of the grand-canonical ensemble of Gibbs is written as^21,35^:

4 $${z}_{1gc}=\sum _{{N}_{i}=0,1}{e}^{-\beta (-{\varepsilon }_{1}-{\mu }_{1}){N}_{i}}=1+{e}^{-\beta (-{\varepsilon }_{1}-{\mu }_{1})}$$

5 $${z}_{2gc}=\sum _{{N}_{i}=0,1}{e}^{-\beta (-{\varepsilon }_{2}-{\mu }_{2}){N}_{i}}=1+{e}^{-\beta (-{\varepsilon }_{2}-{\mu }_{2})}$$

With *z*_*1gc*_ and *z*_*2gc*_ being the partition-functions of the two kinds of sites, *µ*_1_ and *µ*_2_ being the chemical potentials of adsorbed ions and *β* being the Boltzmann factor.

The next step of the statistical physics development comprises the calculation of the average numbers of occupation (*N*_01_ and *N*_02_) of independent [6]Helicene s sites. They are expressed as follows^36,37^:

6 $${N}_{01}=\frac{{H}_{M1}}{1+{e}^{-\beta ({\varepsilon }_{1}+{\mu }_{1})}}$$

7 $${N}_{02}=\frac{{H}_{M2}}{1+{e}^{-\beta ({\varepsilon }_{2}+{\mu }_{2})}}$$

For a classical single-layer adsorption with no disturbances at saturation, the mutual interactions between the adsorbate particles are neglected and then, we use the chemical potential of the ideal gas. Whereas, in the case of saturation level followed with a drop, we use the chemical potential of the real gas in order to introduce the lateral interactions between the adsorbate particles.

Finally, we calculate the analytical expression of the adsorbed quantity (*Q*) corresponding to the single-layer model with two types of sites resulting from the product of the two numbers of ions per sites (*n*_1_ and *n*_2_) and the two average occupation numbers(*N*_01_ and *N*_02_)^21,37^:

8 $$Q={n}_{1}.{N}_{01}+{n}_{2}.{N}_{02}$$

The single-layer model expression established on the basis of the perfect gas (SMPG) is written as:

9 $${Q}_{SMPG}=\frac{{n}_{1}{H}_{M1}}{1+{\left(\frac{{c}_{1}}{c}\right)}^{{n}_{1}}}+\frac{{n}_{2}{H}_{M2}}{1+{\left(\frac{{c}_{2}}{c}\right)}^{{n}_{2}}}$$

Where, n_1_ and n_2_ are the numbers of bonded ions per Helicene site. *H*_*M*1_ and *H*_*M*2_ are the densities of the two spatial conformations *M* and *P* of hexaHelicene. ${c}_{1}=S{e}^{-\beta {\varepsilon }_{1}}$ and ${c}_{2}=S{e}^{-\beta {\varepsilon }_{2}}$ are the energetic parameters.

The expression of the single-layer adsorbed quantity developed on the basis of the real gas law (SMRG) is written as follows:

10 $${Q}_{SMRG}=\frac{{n}_{1}{H}_{M1}}{1+{\left({w}_{1}\frac{1-bc}{c}{e}^{2\beta ac}{e}^{-\frac{bc}{1-bc}}\right)}^{{n}_{1}}}+\frac{{n}_{2}{H}_{M2}}{1+{\left({w}_{2}\frac{1-bc}{c}{e}^{2\beta ac}{e}^{-\frac{bc}{1-bc}}\right)}^{{n}_{2}}}$$

This advanced model presents eight physicochemical parameters: the steric counting parameters *n*_*1*_, *n*_*2*_, *H*_*M1*_ and *H*_*M2*_, the two energetic parameters *w*_1_ and *w*_2_ and the two lateral interactions parameters which are the cohesion pressure *a* and the covolume *b*.

#### L.B.L Double-layers adsorption

The L.B.L double-layers adsorption is shown in the case of silver sulfide isotherms. In order to develop the systematic expression of this model, four energy levels were assumed:

- (-*ε*_1_) characterizes the adsorption of silver ions of the first layer on the first adsorbent type.
- (-*ε*_2_) reflects the adsorption of silver ions on the second spatial conformation of hexaHelicene.
- (-*ε*_3_) is the energy level corresponding to the adsorption of sulfur ions of the second layer on the first spatial conformation.
- (-*ε*_4_) is the energy level of sulfur adsorption on the second adsorbent type.

According to this supposition, the grand-canonical partition function of the L.B.L double-layers model on two types of sites is composed as follows:

11 $${z}_{gc}=\sum _{{N}_{i}=0,1,2}{e}^{-\beta (-{\varepsilon }_{i}-{\mu }_{1}){N}_{i}}=1+{e}^{\beta ({\varepsilon }_{1}+{\mu }_{1})}+{e}^{\beta ({\varepsilon }_{1}+{\varepsilon }_{3}+2{\mu }_{1})}$$

12 $${z}_{gc}=\sum _{{N}_{i}=0,1,2}{e}^{-\beta (-{\varepsilon }_{i}-{\mu }_{2}){N}_{i}}=1+{e}^{\beta ({\varepsilon }_{2}+{\mu }_{2})}+{e}^{\beta ({\varepsilon }_{2}+{\varepsilon }_{4}+2{\mu }_{2})}$$

Following the statistical physics treatment described previously, we deduce the two analytical expressions *Q* of the L.B.L double-layers model with four energy levels:

Based on ideal gas approach:

13 $${Q}_{DMPG}={n}_{1}{H}_{M1}\frac{{\left(\frac{c}{{c}_{1}}\right)}^{n1}+2{\left(\frac{c}{{c}_{3}}\right)}^{2n1}}{1+{\left(\frac{c}{{c}_{1}}\right)}^{n1}+{\left(\frac{c}{{c}_{3}}\right)}^{2n1}}+{n}_{2}{H}_{M2}\frac{{\left(\frac{c}{{c}_{2}}\right)}^{n2}+2{\left(\frac{c}{{c}_{4}}\right)}^{2n2}}{1+{\left(\frac{c}{{c}_{2}}\right)}^{n2}+{\left(\frac{c}{{c}_{4}}\right)}^{2n2}}$$

Based on real gas law:

14 $$\begin{array}{c}{Q}_{DMRG}={n}_{1}{H}_{M1}\frac{{\left(\frac{c}{{w}_{1}(1-bc){e}^{2\beta ac}{e}^{-\frac{bc}{1-bc}}}\right)}^{n1}+2{\left(\frac{c}{{w}_{3}(1-bc){e}^{2\beta ac}{e}^{-\frac{bc}{1-bc}}}\right)}^{2n1}}{1+{\left(\frac{c}{{w}_{1}(1-bc){e}^{2\beta ac}{e}^{-\frac{bc}{1-bc}}}\right)}^{n1}+{\left(\frac{c}{{w}_{3}(1-bc){e}^{2\beta ac}{e}^{-\frac{bc}{1-bc}}}\right)}^{2n1}}+\\ {n}_{2}{H}_{M2}\frac{{\left(\frac{c}{{w}_{2}(1-bc){e}^{2\beta ac}{e}^{-\frac{bc}{1-bc}}}\right)}^{n2}+2{\left(\frac{c}{{w}_{4}(1-bc){e}^{2\beta ac}{e}^{-\frac{bc}{1-bc}}}\right)}^{2n2}}{1+{\left(\frac{c}{{w}_{2}(1-bc){e}^{2\beta ac}{e}^{-\frac{bc}{1-bc}}}\right)}^{n2}+{\left(\frac{c}{{w}_{4}(1-bc){e}^{2\beta ac}{e}^{-\frac{bc}{1-bc}}}\right)}^{2n2}}\end{array}$$

15 $${c}_{1,2,3,4}=S{e}^{-\beta {\varepsilon }_{1,2,3,4}}$$

The coefficients *c*_1,2*,3,4*_ and *w*_1,2,3,4_ are energetic parameters written as function of adsorption energies:

16 $${w}_{1,2,3,4}=S{e}^{-\beta {\varepsilon }_{1,2,3,4}}$$

These two models present common steric parameters: *n*_1_, *n*_2_, *H*_*M1*_ and *H*_*M2*_. Each of them presents four energetic factors which are *c*_*1*_*, c*_*2*_*, c*_3_ and *c*_4_ for the DMPG model and *w*_*1*_*, w*_2_*, w*_*3*_ and *w*_*4*_ for the DMRG model. The DMRG model presents also two Van-der-Waals parameters (the cohesion pressure *a* and the covolume of particles *b*).

#### Numerical results

Numerical fitting program is applied to adjust the four models SMPG, SMRG, DMPG and DMRG with all complexation isotherms.

Note that the results demonstrating that an adsorption isotherm is well fitted with statistical physics model are: the correlation coefficient *R*^2^ tends to the unit, the values of the residual root mean square *RMSE* are less than 3 and the fitting values of the Akaike information criterion *AIC* are the lowest quantities^12,38^.

The values of errors coefficients of adjustment (*R*^*2*^*, RMSE* and *AIC*) are reported in Table 1.

**Table 1:** The values of the correlation coefficient *R*^*2*^, the residual root mean square coefficient *RMSE* and the Akaike information criterion *AIC* deduced from the numerical adjustment of experimental hexa-Helicene adsorption isotherms with the four statistical physics models (SMPG, SMRG, DMPG and DMRG).

Adsorption isotherms		AgCl-[6]Helicene			AgNO_3_- [6]Helicene			Ag_2_S- [6]Helicene		
Temperature(*K*)		293	313	333	293	313	333	293	313	333
SMPG	***R***^***2***^	0.99	0.99	0.98	0.88	0.86	0.89	0.76	0.77	0.71
	***RMSE***	0.04	0.05	0.04	5.2	5.4	6.1	7.2	7.3	8
	***AIC***	6.5	6.9	7	14.2	14.1	13.9	19.3	18.4	19.1
SMRG	***R***^***2***^	0.95	0.93	0.91	0.97	0.99	0.98	0.82	0.84	0.85
	***RMSE***	2.1	2.3	2.2	1.2	2	1.8	6.9	7.1	6.6
	***AIC***	9.9	9.2	9.7	9.6	9.5	9.5	18.2	17.5	17.6
DMPG	***R***^***2***^	0.94	0.94	0.93	0.91	0.91	0.93	0.98	0.96	0.98
	***RMSE***	3.9	4.1	3.8	8.3	7.9	8.2	2.1	3.3	3.2
	***AIC***	13.5	14.2	13.5	15.4	15.6	16	10.2	11	11.1
DMRG	***R***^***2***^	0.91	0.93	0.9	0.94	0.94	0.94	0.95	0.94	0.95
	***RMSE***	3.3	3.8	4	3.2	3.8	3.7	4.4	5.1	5.2
	***AIC***	15.2	14.8	14.7	12.3	13.1	12.9	13.3	14.2	14.5

According to Table 1, it is confirmed that the experimental isotherms of silver chloride are well adjusted by the single-layer adsorption model (SMPG). The SMRG is found in acceptable correlation with the silver nitrate adsorption. The L.B.L double-layers model with four energies (DMPG) is selected for the interpretation of the silver sulfide complexation.

These numerical results are in accordance with the experimental findings given in **section II.3**: silver chloride is confirmed to be the best adsorbate for hexaHelicene complexation because the anionic ions (Cl^−^) do not influence the adsorption mechanism. Moreover, during the silver chloride complexation there are no disturbances at saturation which reflects the highest stability of the formed complexes.

The values of the physicochemical parameters are also deduced through the numerical adjustment. By the intermediate of the models parameters values, the complexation mechanisms can be deciphered in order to select the best adsorbate for [6]Helicene complexation.

### Results of statistical physics modeling

Table 2 indicates the fitting values of all parameters affecting the single-layer complexation of silver chloride and silver nitrate and the double-layers adsorption of silver sulfide.

**Table 2:** Fitting values of physic-chemical variables affecting the single-layer complexation of silver chloride and silver nitrate and the double-layers adsorption of silver sulfide given at 293, 313 and 333 *K*.

Physic-chemical variables	Temperature (*K*)	AgCl-[6]Helicene/SMPG	AgNO_3_- [6]Helicene/SMRG	Ag_2_S- [6]Helicene/DMPG
*n*_1_	**2*93***	0.91	0.71	0.43
	***313***	0.95	0.77	0.45
	***333***	1.01	0.79	0.47
*n*_*2*_	***293***	0.96	0.82	0.49
	***313***	0.94	0.82	0.48
	***333***	0.99	0.85	0.54
*H*_*M1*_ *(µg.cm*^*−2*^)	***293***	50.1	48.3	40.2
	***313***	72.3	59.6	49.7
	***333***	86.8	68.4	55.3
*H*_*M2*_ *(µg.cm*^*−2*^)	***293***	44.6	33.4	22.9
	***313***	53.6	48.1	36.4
	***333***	66.1	59.7	47.7
*a (10*^*−20*^*. J.mL.mol*^*−1*^)	***293***	—	15.3	—
	***313***	—	14.2	—
	***333***	—	12.1	—
*b (10*^*−10*^*. mL.mol*^*−1*^)	***293***	—	23.3	—
	***313***	—	20.1	—
	***333***	—	16.1	—
*c*_1_ *(J.mol*^*−1*^)	***293***	0.006	—	0.0005
	***313***	0.0061	—	0.0005
	***333***	0.0064	—	0.00052
*c*_2_ *(J.mol*^*−1*^)	***293***	0.07	—	0.00081
	***313***	0.072	—	0.00082
	***333***	0.73	—	0.00083
*c*_3_ *(J.mol*^*−1*^)	***293***	—	—	0.049
	***313***	—	—	0.048
	***333***	—	—	0.051
*c*_4_ *(J.mol*^*−1*^)	***293***	—	—	0.053
	***313***	—	—	0.052
	***333***	—	—	0.054
*w*_1_ *(J.mol*^*−1*^)	***293***	—	0.005	—
	***313***	—	0.0052	—
	***333***	—	0.0055	—
*w*_2_ *(J.mol*^*−1*^)	***293***	—	0.064	—
	***3*1*3***	—	0.065	—
	***333***	—	0.065	—

It is necessary to comprehend the physical effect of all models parameters on the adsorption capacities in order to compare the materials used for [6]Helicene complexation with microscopic manner:

- Generally, the higher the value of *n*_1_ or *n*_2_ is, the greater the adsorbed amount is since this parameter represents the number of particles per adsorbent site and it can estimate the accumulation level of silver particles on one site. So, a value of *n* greater than one implies that it is easier to gather many silver particles on one [6]Helicene site. Undoubtedly, the adsorption system with the highest values of *n*_1_ and *n*_2_ is the most appropriate for [6]Helicene complexation in term of reproducibility. According to Table 2, we can clearly see that: *n*_1_(AgCl) > *n*_1_(AgNO_3_) > *n*_1_(Ag_2_S) and *n*_2_(AgCl) > *n*_2_(AgNO_3_) > *n*_2_(Ag_2_S). This means that the silver chloride is the most attractive with the hexaHelicene sites in term of quantity. Besides, all adjusted values of *n*_1_ and *n*_2_ are inferior to one. So it can be concluded that the multi-ionic adsorption into the hexaHelicene sites is impossible because of the electrostatic repulsion interaction between the silver ions. The fitted values of *n*_1_ or *n*_2_ are the lowest for silver sulfide because sulfur ions have an impact on the complexation process. The interaction between the first adsorbed layer of Ag^+^ and the second layer of S^2−^ disfavors the interaction Ag^+^-Helicenes.
- The densities of receptor sites *H*_*M*1_ and *H*_*M*2_ are steric parameters associated with the effectively occupied [6]Helicene sites (*P* and *M*). So, it is evident to note that the adsorbed quantity effectively increases as *H*_*M*1_ and *H*_*M*2_ values increase. As indicated in Table 2, the fitted values of *H*_*M*1_ and *H*_*M*2_ are the highest in the case of the silver chloride adsorption: *H*_*M*1_ (AgCl) > *H*_*M1*_ (AgNO_3_) > *H*_*M1*_ (Ag_2_S) and *H*_*M*2_ (AgCl) > *H*_*M2*_ (AgNO_3_) > *H*_*M2*_ (Ag_2_S). It is noticed that the two types of [6]Helicene sites are more attractive with silver chloride in accordance with the previous investigation of *n*_1_ and *n*_2_. In fact, the chlorine ions have no influence on the complexation procedure so there is a quick penetration of Ag^+^ in the adsorbent pores contrary to the sulfur ions S^−^ which participate at the double-layers adsorption and prevents the Helicenes complexation.
- The cohesion pressure *a* and the covolume *b* are responsible of the instability of the complexes formed during the adsorption of silver nitrate on [6]Helicene. They describe the lateral interactions between the adsorbate particles at free state^10,11,21^. The presence of these parameters in the SMRG model (which depicts the silver nitrate adsorption) indicates high adsorbate-adsorbate interaction. This means that the attractive interaction between the silver ions and the solution is greater than the interaction with the adsorbing surface which leads to a reversible phenomenon at high concentration. Actually, the reversible phenomenon shown in silver nitrate isotherms (Fig. 4) is probably due to the weak binding AgNO_3_- [6]Helicene. This deduct confirm the choice of silver chloride as best material for Helicenes adsorption but it requires confirmation through energetic investigation. Furthermore, we can see from Table 2 that the Van-der-Waals parameters *a* and *b* decline with ascending of temperature. This result indicates that the lateral interactions between the adsorbates and the disturbances during the complexation process are the lowest at high temperature. This leads to estimate that the silver nitrate complexation can be carried out if we increase the temperature. This fact cannot be achieved by means of the QCM setup because the gold electrode of quartz crystal is very sensitive to high temperatures.
- The adsorption energy calculation is the most significant factor frequently applied to characterize the interaction type of adsorption system. The single layer model SMPG evolves two energetic parameters *c*_1_ and *c*_2_. Thus the complexation energies (−*ΔE*_1_) and (−*ΔE*_2_) which correspond to the complexation of silver chloride on the two spatial conformations of hexaHelicene are calculated by the following relation:

17 $$-\Delta {E}_{1,2}=RT\ast \mathrm{ln}\,({c}_{1,2}/S(AgCl))$$

where, *R* is the ideal gas constant (8.3144621 *J mol*^*−*1^ *K*^*−1*^), *T* is the absolute temperature, *S*(AgCl) is the solubility of investigated adsorbate (AgCl) and *c*_*1*_ and *c*_2_ are the fitted value energetic parameters deduced from the adjustment with SMPG Model.

The SMRG model gives rise to two energetic parameters *w*_*1*_ and *w*_*2*_. Then, the complexation energies (−*ΔE*_*1*_) and (−*ΔE*_*2*_) of silver nitrate on the two kinds of hexaHelicene are calculated by the subsequent relation:

18 $$-\Delta {E}_{1,2}=RT\ast \mathrm{ln}({w}_{1,2}/S(AgN{O}_{3}))$$

With, *S*(AgNO_3_) is the solubility AgNO_3_. The fitted value of the energetic parameters *w*_*1*_ and *w*_*2*_ are deduced from the adjustment of experimental isotherms with the SMRG Model.

The L.B.L double-layers adsorption of silver sulfide offers four adsorption energies by the intermediate of the DMPG model. They are calculated by the following formula:

19 $$-\Delta {E}_{1,2,3,4}=RT\ast \mathrm{ln}({c}_{1,2,3,4}/S(Ag{S}_{2}))$$

With *S* (AgS_2_) is the silver sulfide solubility.

The adsorption energies of the three complexation systems determined at three temperatures are given in Table 3.

**Table 3:** Estimated values of adsorption energies of the three complexation systems determined at three temperatures (293–333 *K*).

Molar adsorption energy (*kJ.mol*^*−1*^)	Temperature (*K*)	AgCl-[6]Helicene	AgNO_3_-[6]Helicene	Ag_2_S-[6]Helicene
*|*−*ΔE*_*1*_*|*	**2*9*3**	88.4	43.6	33.6
	***313***	95.4	48.6	39.4
	***333***	111.2	50.7	44.1
*|*−*ΔE*_*2*_*|*	***293***	77.9	38.4	29.4
	***313***	91.2	40.2	33.2
	***333***	98.4	49.6	38.4
*|*−*ΔE*_*3*_*|*	***293***	—	—	21.3
	***313***	—	—	18.4
	***333***	—	—	16.5
*|*−*ΔE*_4_*|*	***293***	—	—	20.4
	***313***	—	—	18.5
	***333***	—	—	16.2

For silver sulfide adsorption, it should be noted that (−*ΔE*_*1*_) and (−*ΔE*_*2*_) reflects the silver particles adsorption at the level of the first adsorbed layer i.e. they characterize the Ag^+^- [6]Helicene interaction. However, (−*ΔE*_3_) and (−*ΔE*_4_) are related to the adsorption of the second layer of sulfur ions^11,26^. Therefore, it is clear that (−*ΔE*_3_) and (−*ΔE*_4_) are not useful for the comparison of the formed complexes and then, the choice of the best adsorbate for hexaHelicene complexation depends on the comparison of (−*ΔE*_1_) and (−*ΔE*_2_) of the three systems.

In view of Table 3, the adsorptions energies are ordered as follows:

|(−*ΔE*_1_) | (AgCl) > |(−*ΔE*_1_) | (AgNO_3_) > |(−*ΔE*_1_) | (Ag_2_S) and | (−*ΔE*_2_) | (AgCl) > |(−*ΔE*_2_) | (AgNO_3_) > |(−*ΔE*_2_) | (Ag_2_S). This energetic order shows that the interaction AgCl - [6]Helicene is the most elevated and then, silver chloride is the best complexed material in term of stability and applicability.

Moreover, the values | (−*ΔE*_1_) | (AgNO_3_), |(−*ΔE*_1_) | (Ag_2_S), |(−*ΔE*_2_) | (AgNO_3_) and | (−*ΔE*_2_) | (Ag_2_S) are found inferior to 80 kJ mol^−1^. Therefore, the silver nitrate and the silver sulfide adsorptions take place via reversible physical process. Generally, these binding are carried out via hydrogen bonds or Van der Waals forces^11,12^. On the other hand, the chemical bonding forces are shown in the case of silver chloride- [6]Helicene since | (−*ΔE*_1_) | (AgCl) and | (−*ΔE*_2_) | (AgCl) are higher than 80 kJ mol^−1^^26^.

In order to check the nature of silver- [6]Helicene interaction, a DFT simulation is proposed in the next section.

### Density functional theory (DFT) calculation and results

In order to elaborate the complexation characteristics of silver on hexaHelicene, theoretical calculations were carried out at the density functional level of theory (DFT, B3LYP functional)^26,39^, employing the Gaussian 09 suite of programs^39^. The LanL2DZ basis set was utilized, and the optimizations were unconstrained. DFT simulations were performed to estimate the bond distances associated with the silver metal adsorption and the subsequent binding energies of the formed complex.

**Figure 5:**
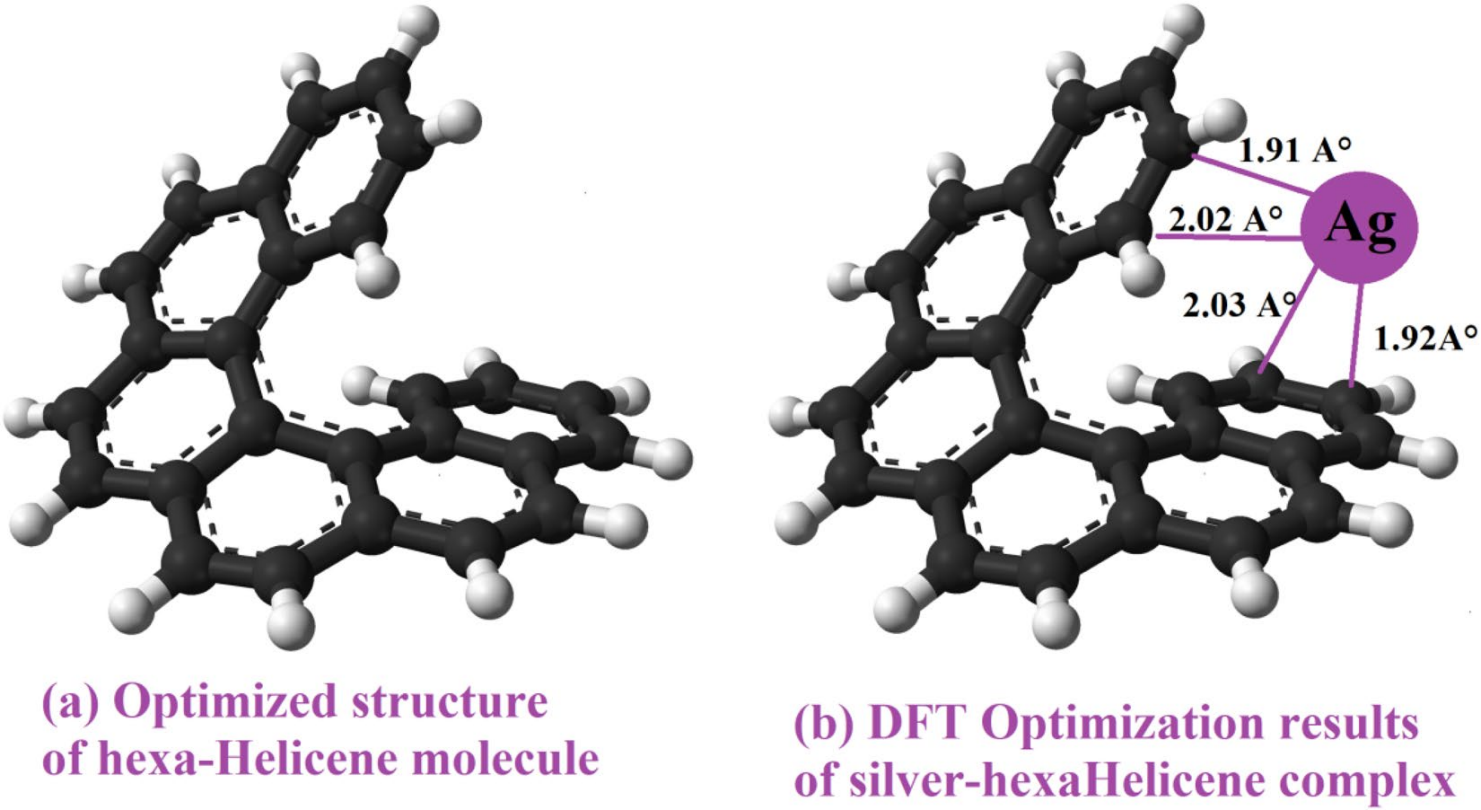
Density functional theory (DFT) optimization: **(a)** Proposed structure of hexa-Helicene used in DFT calculation and **(b)** Results of the DFT simulation of silver-hexaHelicene complex.

Actually, The proposed structure of the silver- [6]helicene complex was anticipated on the basis of the profound conformational analysis i.e., six distinctive mutual positions of the [6]Helicene ligand and the cationic silver were considered during the geometry optimization^40,41^. In the model calculations, we optimized the molecular geometries of the free [6]helicene ligand and its complex with silver particle. The structure of the mentioned [6]helicene used in DFT calculation is depicted in Fig. 5a.

The interaction energy, *E*(int) for the connections between the silver particles and the adsorbent surface of hexaHelicene was determined using the next expression^26,40,41^:

20 $$E(\mathrm{int})=E(complex)-(E(Ag)+E(hexaHelicene))$$

where, *E* (complex) is the pure electronic energy of the formed complex Ag- [6]helicene, *E*(Ag) is the estimated energy for the silver metal and *E*(hexaHelicene) is the energy of the molecular structure of the [6]helicene surface.

Results of DFT simulations are illustrated in Fig. 5b.

From Fig. 5b, in the resulting Ag- [6]helicene complex, the silver metal is bound by four relatively strong bonds to four carbon atoms from the two terminal benzene rings of the parent ligand via cation-π interaction. The bonds lengths ranged from 1.91 *A°* to 2.03 *A°*. In addition, we note an interaction energy value of −98.3 *kJ/mol* which also confirms the formation of this complex. The estimated binding energy corresponded to covalent bonds which could be associated to chemisorption mechanism. This finding is consistent with the results obtained with the SMPG adsorption model (silver chloride adsorption) based on statistical physics approach.

Consequently, the DFT investigation has proven that high interaction takes place during the adsorption of silver chloride on hexaHelicene.

## Conclusion and Recommendation

The aim of this work was to describe a cancer drug at the ionic scale. The ultimate result is that hexaHelicene can function as sensor of the cationic silver metal. Overall, despite all experimental isotherms are carried out by the intermediate of the QCM method and describe the same chemical process ([6]Helicene complexation), three different phenomena are observed. The adsorption of silver chloride is explained via the single-layer model on two types of sites SMPG, the silver nitrate adsorption is discussed by the intermediate of the SMRG model which takes into account the lateral interaction between the particles and the silver sulfide isotherms are in good correlation with the L.B.L adsorption model DMPG.

The patterns of the three complexation frameworks are discussed by comparing the fitted parameters of the three advanced statistical physics models. The parameters numbers of silver ions per Helicene site (*n*_1_ and *n*_2_) demonstrated that the silver chloride is the best interactive material with hexaHelicene sites in term of quantity and that no accumulation occurs on one receptor site during the complexation mechanisms (*n*_*1,2*_ < 1). The densities of occupied sites *H*_*M1*_ and *H*_*M2*_ indicated that the two types of [6]Helicene sites *P* and *M* are more attractive with the silver chloride. The calculation of the complexation energies and the DFT optimizations confirm the highest stability of the AgCl-[6]Helicene complex. Actually, the interaction AgCl-[6]Helicene is found typical to a chemisorption process.

Correlating all QCM measurements and statistical physics discussions, we recommend the use of silver chloride as adsorbate material for a modern application of Helicenes complexation.

### Comparison with similar works

Theoretically speaking, similar previous works on [7]Helicene (e.g. lithium-heptaHelicene and Potassium-heptaHelicene…)^10,21^ showed that the presence of chlorine ions has no impact on the complexation process which is in accordance with the case of silver chloride. However, the mentioned previous complexes are explained by a monolayer model that presents disturbances at saturation indicating that the complexes formed with heptaHelicene are unstable like the case of silver nitrate in the present investigation. This confirms that the use of hexaHelicene as complexing adsorbent of alkali metals is more successful and conceptive than the use of heptaHelicene. Moreover, despite the silver sulfide-hexaHelicene was found a stable complex, the L.B.L adsorption on Helicenes molecules is shown at the first time in this investigation. Thus, past works have demonstrated that the adsorption of cationic metals (Mg^2+^, Zn^2+^, and Fe^2+^…) on porphyrins was carried out on the basis of the layer by layer approach in the case of nitrate ions presence^11,12^. So, we can notice that an L.B.L adsorption on Helicenes can be only shown in the case of using an adsorbate involving sulfur ions because silver nitrate complexation resulted in a single-layer adsorption which indicated that the nitrate ions were not involved in the adsorption mechanism.
